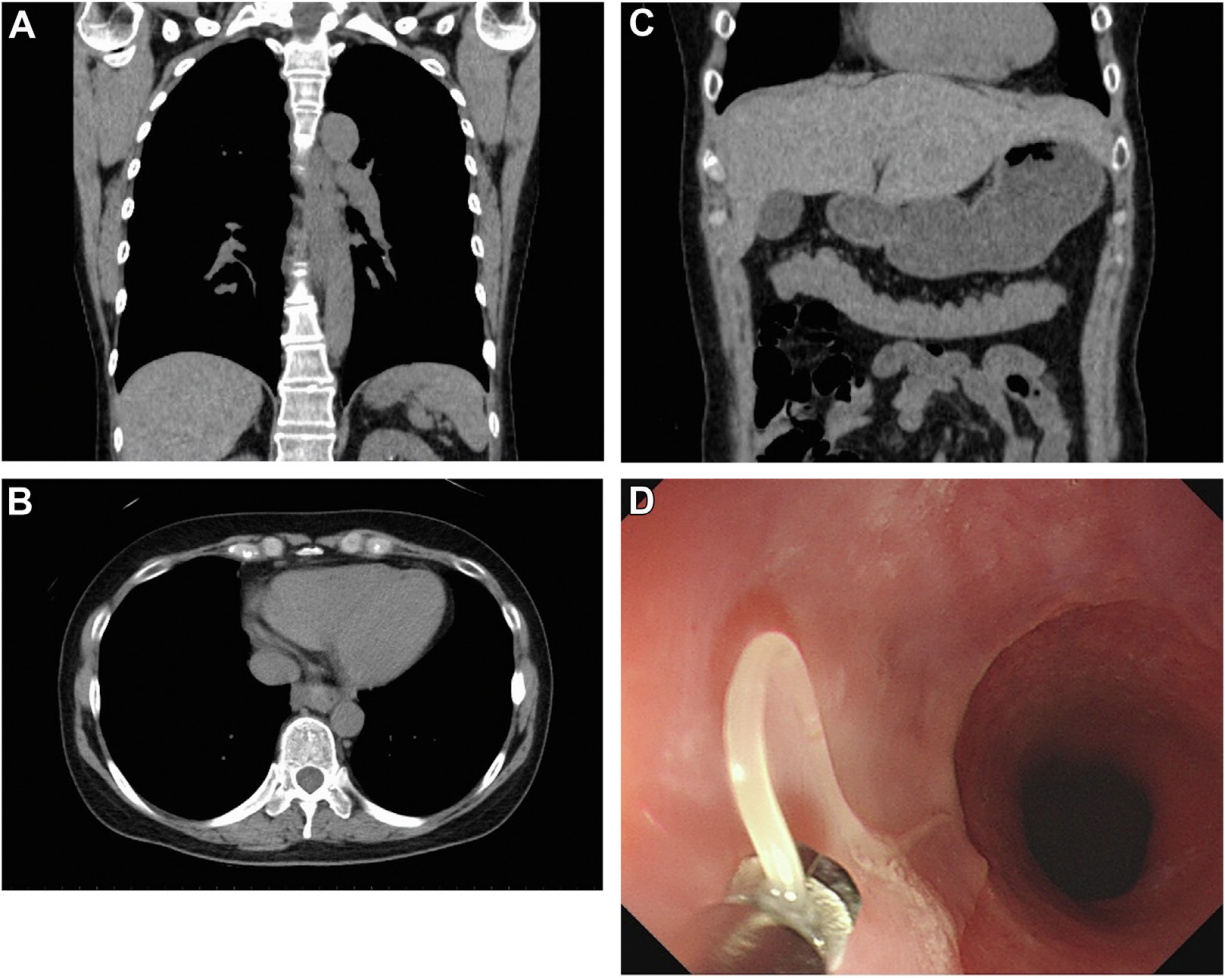# Unusual Cause of Recurrent Episodes of Dysphagia

**DOI:** 10.1016/j.gastha.2023.07.007

**Published:** 2023-07-18

**Authors:** Yasutoshi Shiratori, Ryosuke Yokosuka, Katsuyuki Fukuda

**Affiliations:** Department of Gastroenterology, St. Luke’s International Hospital, Tokyo, Japan

A 59-year-old woman came to our emergency department with dysphagia. The patient ate a steak course dinner. She had the same history one year ago, and a follow-up endoscopy one month later at that time showed no apparent abnormalities. She was afebrile, and her vital signs were stable. Blood exam revealed mild elevation of C-reactive protein (0.50 mg/dL). Plain chest computed tomographic scan presented broad thickening of the esophageal wall (Figure A) and fluid correction next to the esophagus (Figure B). Gastric wall was unremarkable (Figure C). Urgent esophagogastroduodenoscopy did not reveal residual food or stricture in the esophagus. Instead, anisakis larvae were detected invading the lower esophagus (Figure D). After removing it using forceps, symptoms were rapidly resoluted. The anisakis was presumed to be in the raw fish of the steak dinner appetizer, and the previous event also occurred after the Japanese sushi dinner. The stomach is the most common site of infection, with infections in the esophagus being very rare (1.4%). Although gastric anisakiasis is mainly characterized by epigastric pain and vomiting, esophageal anisakiasis presents with dysphagia. The esophageal wall is relatively thin, with a risk of mediastinitis in esophageal anisakiasis; therefore, urgent esophagogastroduodenoscopy should be performed.

**Figure:**